# Extreme weather events in developing countries and related injuries and mental health disorders - a systematic review

**DOI:** 10.1186/s12889-016-3692-7

**Published:** 2016-09-29

**Authors:** Elisabeth Rataj, Katharina Kunzweiler, Susan Garthus-Niegel

**Affiliations:** 1 Center for Evidence-based Healthcare, University Hospital Carl Gustav Carus, TU Dresden, Germany; 2 Cochrane Germany, Medical Center - University of Freiburg, Faculty of Medicine, University of Freiburg, Freiburg, Germany; 3 Institute and Policlinic of Occupational and Social Medicine, University Hospital Carl Gustav Carus, TU Dresden, Germany; 4 Department of Child Health, Norwegian Institute of Public Health, Oslo, Norway; 5 Institute and Outpatient Clinics of Psychotherapy and Psychosomatic, University Hospital Carl Gustav Carus, TU Dresden, Germany

## Abstract

**Background:**

Due to climate change, extreme weather events have an incremental impact on human health. Injuries and mental health disorders are a particular burden of disease, which is broadly investigated in high income countries. Most distressed populations are, however, those in developing countries. Therefore, this study investigates mental and physical health impacts arising from extreme weather events in these populations.

**Method:**

Post-traumatic Stress Disorder (PTSD), injury [primary outcomes], anxiety and depressive disorders [secondary outcomes], caused by weather extremes were systematically analyzed in people of developing countries. A systematic review of observational studies was conducted searching six databases, complemented by hand search, and utilizing two search engines. Review processing was done independently by two reviewers. Prevalence rates were analyzed in a pre/post design; an additional semi-structured search was conducted, to provide reference data for studies not incorporating reference values.

**Results:**

All 17 identified studies (70,842 individuals) indicate a disease increase, compared to the reference data. Increase ranges from 0.7–52.6 % for PTSD, and from 0.3–37.3 % for injury. No studies on droughts and heatwaves were identified. All studies were conducted in South America and Asia.

**Conclusion:**

There is an increased burden of psychological diseases and injury. This finding needs to be incorporated into activities of prevention, preparedness and general health care of those developing countries increasingly experiencing extreme weather events. There is also a gap in research in Africa (in quantity and quality) of studies in this field and a predominant heterogeneity of health assessment tools.

PROSPERO registration no.: CRD42014009109

**Electronic supplementary material:**

The online version of this article (doi:10.1186/s12889-016-3692-7) contains supplementary material, which is available to authorized users.

## Background

Weather related issues, most of all climate change, have risen to the top of the international environment agenda in the last decades. Sea level rise and weather phenomena are of increasing research interest. The intersections of weather extremes and health are not investigated in depth, particularly not in developing countries. There, about 32 million people fled their homes, just in 2012, because of extreme weather events [1]. People in developing countries carry a double burden of deprivation since they are more vulnerable to the effects of environmental degradation plus they have to cope with the threat to their immediate environment and health.

The Intergovernmental Panel on Climate Change defines weather extremes as abnormal events which, in comparison to similar events, differ in average and have a very irregular period of repetition [2]. Climate change increases the likelihood of extreme weather events which have more than doubled in the past decades [3]. Floods, droughts, storms, and heatwaves are the events the leading literature refers to as the most common and most important disasters [3–5]. Post-disaster research has widely been conducted in Western populations. Very little research has been done on developing countries. Even though data on injury might be comparatively easily obtained and monitored, e.g. via death statistics or hospitalization rates, there still remains a huge shortcoming in the current state of research in this field [4, 8]. Furthermore, several other publications highlight the research gap on post disaster mental health outcomes, like anxiety and depression disorders [4, 6–8, 24]. These are expected to induce a severe burden of disease; they are assumed to be potentially large but under-examined, underestimated and not adequately monitored.

This long-term psychological morbidity is reported to be one of the main adverse effect of weather disasters [4]. The mental health situation may also be directly connected to the event, as in PTSD. This shift from initial impacts of the emergency to the phase of dealing with long-term health issues needs to be analyzed carefully.

Against this background this study’s leading research question is: How is the mental and physical health status of people in developing countries affected by extreme weather events?

## Methods

This study closely adheres to the reporting guideline for systematic reviews (PRISMA), its’ protocol was a priori registered (26.03.2014; PROSPERO) [9]. A completed PRISMA checklist is provided as a supplementary file (see Additional file 1).

### Eligibility criteria

Eligibility is defined by using the PICOS scheme.

### Participants

The study populations are countries with low, medium, and high human development derived from the 2013 Human Development Report [10]. The Human Development Index (HDI) is a comparative measure which all countries fall into four development categories. The term “developing countries” is used throughout this study for the three lowest included HDI categories.

### **I**ntervention (exposure)

Flood, drought, storm and heatwave are eligible exposures. The definitions are derived from the American Meteorological Society’s glossary [11].

### Comparator

People in developing countries who did not experience extreme events are the comparison group; alternatively population data from prior to the event are used.

### Outcome

PTSD and injury are defined as primary outcomes. Anxiety and depressive disorders are secondary outcomes. DSM-IV [12] and ICD 10 [13] are jointly the basis for included indications.

### Study design

Eligible for this analysis are observational studies, comprising case control, cohort and cross-sectional studies. Only published studies are included.

No start was set for the search, it ended in April 2014. Languages included are limited to English and German.

### Information sources

The included studies were identified by searching electronic databases, hand searching reference lists and relevant journals, plus consulting two search engines. The search was applied to Medline and Embase via Ovid, as well as Web of Science (Core Collection) and PsycINFO via EBSCOhost. CAB Direct was searched directly and PILOTS via ProQuest. Hand search was conducted in *Global Environmental Change* (Elsevier) and *Climatic Change* (Springer Link). Google Scholar and the WHO’s Virtual Health Library (VHL) were searched.

### Literature search

An initial search strategy was developed for Medline (see Additional file 2) and adapted for the other databases (according to each data basis’ individual search requirements). This search string was developed by identifying study protocols from the *Cochrane Database of Systematic Reviews*; each search string sequence was supported by a published protocol on an equivalent topic. For example, for the population, defined as people living in developing countries, an applicable protocol was identified [14]. That protocol’s search strings section on *developing countries* served as basis for designing the search string, and so forth. The sequence of *extreme weather events* was developed and pre-tested by the authors. Search filter for observational studies were adopted from SIGN [15].

### Study selection process

The selected sources were searched and duplicates removed. The screening of titles and abstracts was performed in a double blinded manner (ER, KK). The inter-rater agreement was pre-tested on 50 studies, with no disagreement (*k* = 1; percentage agreement = 100 %) with the software *R* (packages *irr* and *psych*). The assessment of eligibility and the full-text screening were independently conducted (ER, KK).

### Data items and collection process

Data collection forms were pilot-tested and refined. Data items were harmonized with the recommended checklist [16]. Information was excerpted on: eligibility, method, participants and setting, exposure, outcomes, results, and other information. Data were extracted in duplicate (ER, KK).

### Risk of bias in individual studies

To ascertain validity of each eligible observational study two reviewers (ER, KK) independently determined the selection of the study population and comparability as well as the exposure (for case control studies) or the outcome (for cohort and cross sectional studies). Risk of bias was assessed by using the *Newcastle - Ottawa Quality Assessment Scale* (NOS) which is provided for case control and cohort studies [17]. The scale for cross sectional studies was derived from an analysis by Herzog et al. [18]. Rating rules were set a priori and the assessment pre-tested on one study of each included study design. For example, a sample size was rated as “not justified”, if less than 100 individuals were included, and the non-responder analysis was judged to be “satisfactory”, if the response-rate reached at least 80 %. Accordingly, the study quality is presented by means of the overall risk of bias in percentages.

### Summary measures

Primary outcome measures are 12-month prevalence rates, which are presented tabularly. To compare and interpret post-disaster prevalence rates, reference data (if not reported) were additionally searched, covering relevant WHO sources [19, 20]. Search terms were the outcome, year and country. This pre/post analysis is visualized in histograms according to each outcome. Additionally, the global prevalence rates are reported. Different study types are not combined, but differences between results compared. Different disaster types and the findings in children and adults are not combined, as recommended [21, 22].

### Synthesis of the results

As stated a-priori in the review’s study protocol, a meta-analysis may be conducted, if feasible. However, comparators, time points, and measuring tools vary strongly throughout the studies, revealing a high degree of study heterogeneity. Therefore, the requirements for conducting a meta-analysis [16] are not fulfilled and thus not indicated for this review. The analysis of the included studies is conducted in a descriptive and comparative way.

## Results

### Study selection

**Fig. 1:**
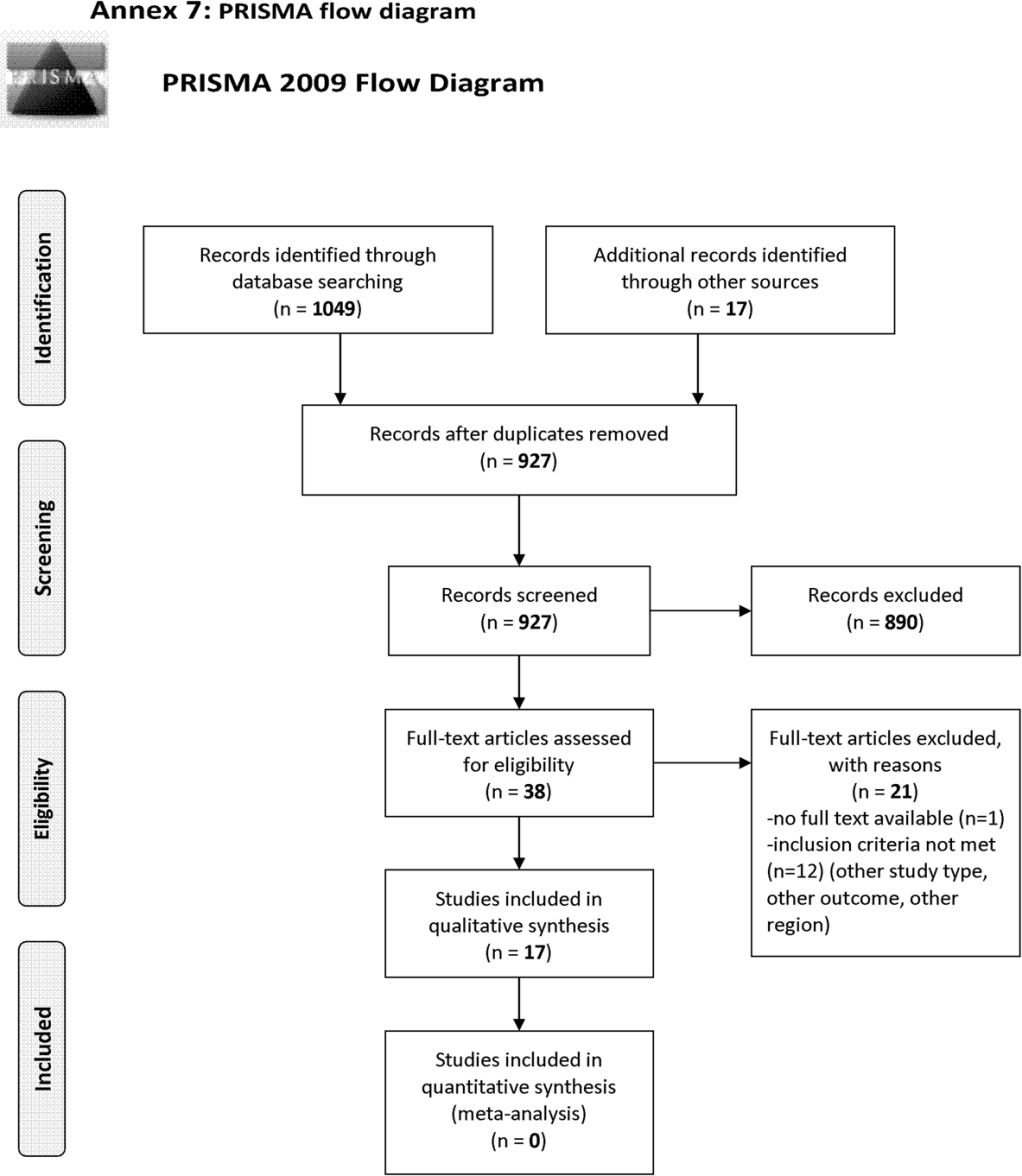
PRISMA flow chart

The search was conducted and duplicates removed, 927 reports remained (Fig. 1). The screening process was pre-tested and conducted independently (ER, KK). The inter-rater reliability of the title/abstract screening displays excellent agreement (*k* = 0.85; percentage agreement: 99.1 %) [23]. 38 studies were carried on for full-text screening; subsequently 21 studies were excluded for not meeting the inclusion criteria.

### Study characteristics

For each of the 17 included studies characterizing data were extracted (ER) and checked (KK) (Table 1).

**Table 1:** Summary of included studies

Study, author/year	Study design	Country (HDI ^a^)	Event	Year	Outcomes	N
Amstadter et al. 2009 [24]	Cohort	Vietnam (medium)	Storm, Typhoon Xangsane	2006	PTSD, MDD, GAD	797
Bich et al. 2011 [42]	Cross sectional	Vietnam (medium)	Flood	2008	Injury	871
Biswas et al. 2010 [43]	Cross sectional	Bangladesh (low)	Flood	2007	Injury	638 women
Caldera et al. 2001 [26]	Cross sectional	Nicaragua (medium)	Storm, Hurricane Mitch	1998	PTSD	496
Goenjian et al. 2001 [25]	Cross sectional	Nicaragua (medium)	Storm, Hurricane Mitch	1998	PTSD, Depression	158 students
Huang et al. 2010 [27]	Cross sectional	China (medium)	Flood	1998	PTSD	25,478
Kar et al. 2004 [33]	Cross sectional	India (medium)	Storm, super-cyclone	1999	PTSD, Anxiety, Depression	540
Kar & Bastia 2006 [34]	Cross sectional	India (medium)	Storm, super-cyclone	1999	PTSD, MDD, GAD	108 students
Kar et al. 2007 [30]	Cross sectional	India (medium)	Storm, super-cyclone	1999	PTSD	447 students
Kohn et al. 2005 [44]	Cross sectional	Honduras (medium)	Storm, Hurricane Mitch	1998	PTSD, Depression	800
Norris et al. 2006 [28]	Cross sectional	Mexico (high)	Flood due to storm	1999	PTSD	666
Patrick & Patrick 1981 [38]	Cross sectional	Sri Lanka (high)	Storm, cyclone	1978	Anxiety, Depression	171
Simeon et al. 1993 [37]	Cohort	Jamaica (high)	Storm, Hurricane Gilbert	1988	Injury	125 children
Sjöberg & Yearwood 2007 [45]	Cross sectional	Grenada (high)	Storm, Hurricane Ivan	2004	Injury	185
Sugimoto et al. 2011 [32]	Cohort	Bangladesh (low)	Storm, tornado	2005	Injury	35,225
Wu et al. 2011 [29]	Cross sectional	China (medium)	Storm, snowstorm	2008	PTSD	968 students
Xu et al. 2012 [31]	Cross sectional	China (medium)	Storm, snowstorm	2008	Injury	3169

*PTSD* post-traumatic stress disorder, *MDD* major depressive disorder, *GAD* general anxiety disorder

Explanation: ^a^ Human Development Index category

A total of 70,842 individuals are included. Six studies reported events in South America; the majority is from Asia (eleven studies). No reports from Africa were identified.

Forty three thousand one hundred eighty nine individuals experienced a storm (tropical cyclone, hurricane, tornado, snowstorm). 27,653 individuals experienced floods. No reports on heatwaves and droughts were identified.

Twenty-nine different assessment tools were used and the time points of measuring reached from a few days up to two years post-disaster. Data were acquired via clinical examination, interview, and questionnaire.

Only four studies reported a comparison group. Another two of the 17 studies compared the prevalence rates with pre-existing data. Three studies analyzed rates in populations exposed to different extent (low/medium/high or low/high). Seven studies exclusively reported prevalence rates of one exposed group and one study developed a prediction model.

### Risk of bias within studies

**Fig. 2:**
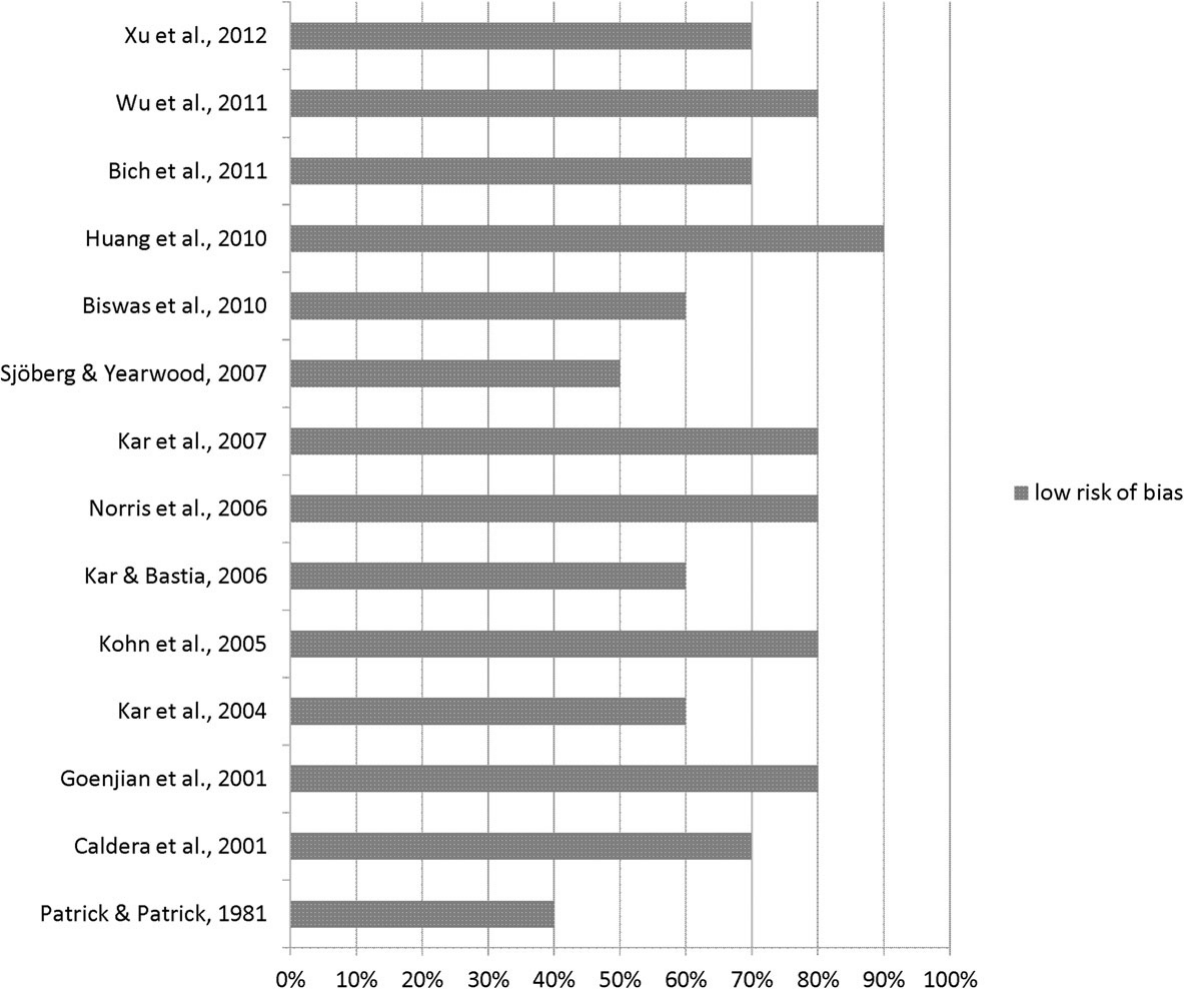
Risk of bias in cross sectional studies

**Fig. 3:**
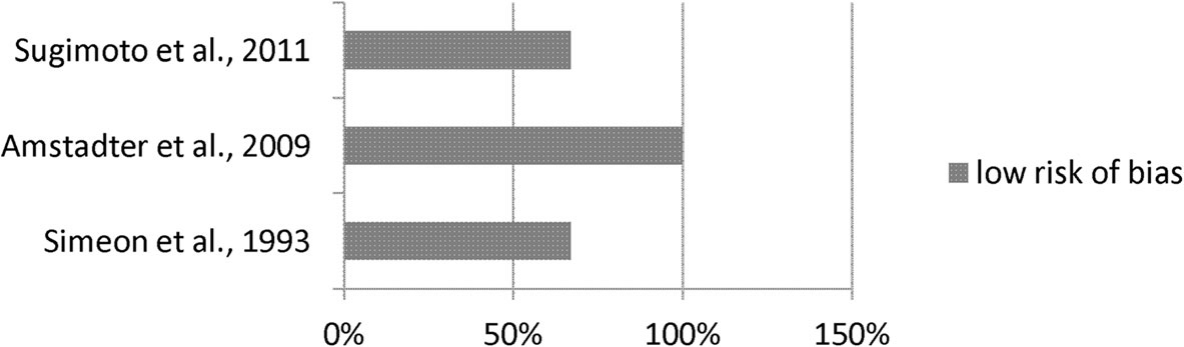
Risk of bias in cohort studies

The assessment tool NOS is interpreted both as checklist and as scale [17]. It was independently applied (ER, KK) with near-perfect inter-rater agreement (*k* = 0.86; percentage agreement: 91 %) [23]. Accordingly the study quality is visualized (Figs. 2 and 3). In using NOS as the checklist the categories: *selection*, *comparability*, and *outcome* were assessed.

### Effects of the exposure

**Fig. 4:**
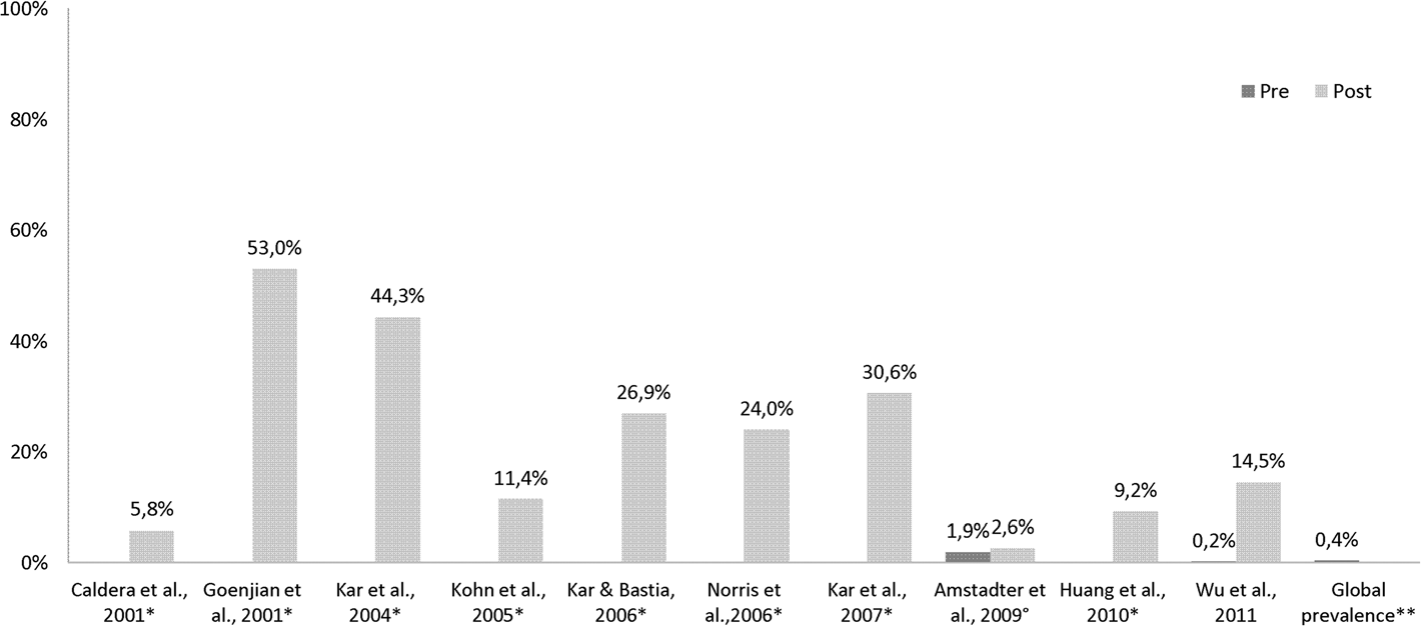
Pre/post analysis of PTSD prevalence in cross sectional and cohort° studies (*data not available; **according to GBD [19])

**PTSD** post-disaster prevalence rates are presented in Table 2. For nine studies no reference data were identified, thus the global prevalence rate (0.37 % [19]) is reported (Fig. 4). Each of the ten studies report an increase in PTSD compared to the global rate.

**Table 2:** Results of PTSD prevalence rates in individual studies (total *n* = 30,458)

Study (author/year)	Country (HDI^a^)	Event/year	N	Assessment tool	Time point measured	PTSD prevalence
Cross sectional studies						
Caldera et al. 2001 [26]	Nicaragua (medium)	Storm, Hurricane Mitch, 1998	496	Harvard Trauma Questionnaire (HTQ)	6 months post	5.8 %
Goenjian et al. 2001 [25]	Nicaragua (medium)	Storm, Hurricane Mitch, 1998	158 students	Child Posttraumatic Stress Reaction Index (CPTS-RI)	6 months post	90 %, 55 %, 14 %^b^
Huang et al. 2010 [27]	China (medium)	Flood, 1998	25,478	Questionnaire	24 months post	9.2 %
Kar et al. 2004 [33]	India (medium)	Storm, super-cyclone, 1999	540	Post traumatic symptom scale (PSS) & Self-Reporting Questionnaire (SRQ)	5 months post	44.3 %
Kar & Bastia 2006 [34]	India (medium)	Storm, super-cyclone, 1999	108 students	Clinical examination & Mini international Neuropsychiatric Interview for children/adolescents (MINI-KID)	14 months post	26.9 %
Kar et al. 2007 [30]	India (medium)	Storm, super-cyclone, 1999	447 students	Clinical examination & ICD-10-symptom check-list & semi-structured questionnaire	12 months post	30.6 %
Kohn et al. 2005 [44]	Honduras (medium)	Storm, Hurricane Mitch, 1998	800	Composite International Diagnostic Interview Schedule (CIDI); Impact of Event Scale (IES)	2 months post	8.9 %, 11.6 %, 13.6 %^c^
Norris et al. 2006 [28]	Mexico (high)	Flood due to storm, 1999	666	Modified version of CIDI	6 months post	24 %
Wu et al. 2011 [29]	China (medium)	Storm, snowstorm, 2008	968 students	IES (revised version)	3 months post	14.5 %
Cohort study						
Amstadter et al. 2009 [24]	Vietnam (medium)	Storm, Typhoon Xangsan, 2006	797	Pre: SRQ; Post: National Women’s Study PTSD Module	3 months post	2.6 %

Explanation: ^a^Human Development Index category; ^b^3 differently affected cities; ^c^3 age groups

The applied instruments covered two disaster-specific ones (Harvard Trauma Questionnaire (HTQ), Impact of Event Scale (IES)) and some that do not link psychiatric symptoms to an experienced event (e.g. Self-Reporting Questionnaire (SRQ), Composite International Diagnostic Interview Schedule (CIDI)); two child-specific instruments were applied (Child Posttraumatic Stress Reaction Index (CPTS-RI (CPTS-RI) and Clinical examination & Mini international Neuropsychiatric Interview for children/adolescents (MINI-KID)) (Table 2).

Several studies additionally investigated predictive and risk factors for PTSD. These were: prior traumatic events or mental health problems [24–29], high disaster exposure [24, 25, 28, 30], death of a relative or witnessing someone die [25–27, 30], low or no education [26–28, 30], female sex [26–28], and destruction of the house [26, 30].

**Fig. 5:**
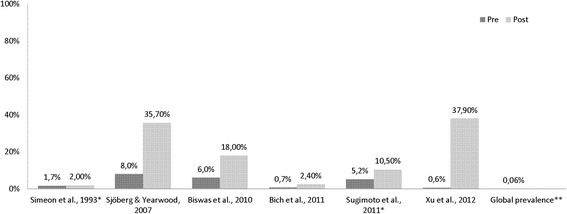
Pre/post analysis of injury prevalence in cross sectional and cohort* studies (** according to [46])

**Injury** prevalence rates were assessed (Table 3) and compared to the reference data. Each of the six studies that investigated injuries indicates an increase in the individual prevalence rates (Fig. 5).

**Table 3:** Results of injury prevalence rates in individual studies (total *n* = 40,213)

Study (author/year)	Country (HDI^a^)	Event/year	N	Assessment tool	Time point measured	Injury prevalence
Cross sectional studies						
Bich et al. 2011 [42]	Vietnam (medium)	Flood, 2008	871	Structured interview & data of MICRODIS household survey	1 month post	Exposed: 2.4 %; Control: 0.7 %
Biswas et al. 2010 [43]	Bangladesh (low)	Flood, 2007	638 women	Face to face interview, semi-structured questionnaire	Few days post	18 %
Sjöberg & Yearwood 2007 [45]	Grenada (high)	Storm, Hurricane Ivan, 2004	185	Hospital records	1 month post	35.7 % (women, *n* = 16, men, *n* = 50)
Xu et al. 2012 [31]	China (medium)	Storm, snowstorm, 2008	3169	Structured questionnaire	Few days post	37.9 %
Cohort studies						
Simeon et al. 1993 [37]	Jamaica (high)	Storm, Hurricane Gilbert, 1988	125 children	Structured questionnaire	2–4 months post	Exposed: 1.7 %, 1.8 %, 2.4 % ^b^; Control: 1.3 %, 2.1 %^c^
Sugimoto et al. 2011 [32]	Bangladesh (low)	Storm, tornado, 2005	35,225	Interview	4 months post	10.5 %

Explanation: ^a^Human Development Index category; ^b^3 2-month-periods during/post-disaster; ^c^2 2-month-periods, pre-disaster

Two studies investigated the following risk factors for being injured during an extreme event: age above 45 years, female sex [31, 32], being outdoors, destruction of the house, tin construction materials [32].

**Fig. 6:**
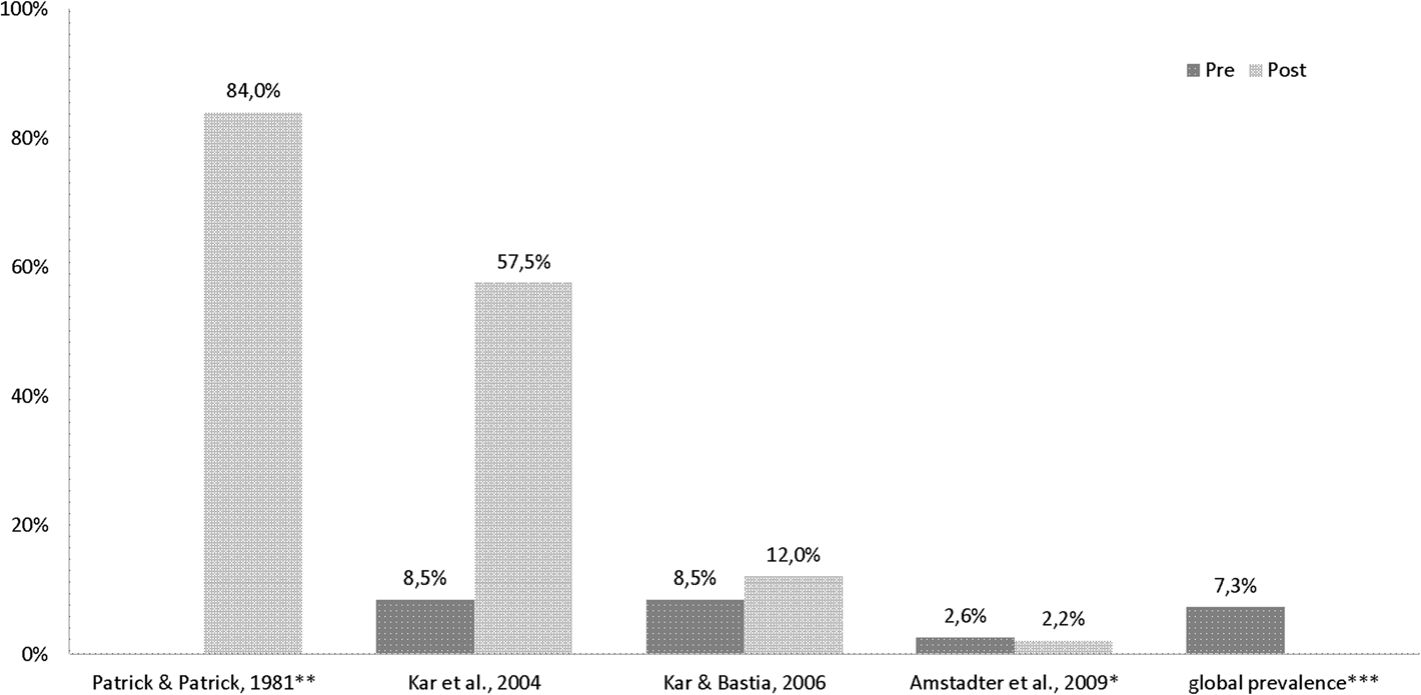
Pre/post analysis of anxiety prevalence in cross sectional and cohort* studies (**data not available; ***according to [47])

**Anxiety disorder** rates (Table 4) were assessed and analyzed. With the exception of one study [24] all post-disaster prevalence rates were higher than those of the non-exposed (Fig. 6).

**Table 4:** Results of anxiety prevalence rates in individual studies (total *n* = 1616)

Study, author/year	Country (HDI^a^)	Event/year	N	Assessment tool	Time point measured	Anxiety prevalence
Cross sectional studies						
Kar et al. 2004 [33]	India (medium)	Storm, super-cyclone, 1999	540	Hospital Anxiety and Depression Scale (HADS) & SRQ	5 months post	57.5 %
Kar & Bastia 2006 [34]	India (medium)	Storm, super-cyclone, 1999	108 students	Clinical examination & MINI-KID	14 months post	12 %
Patrick & Patrick 1981 [38]	Sri Lanka (high)	Storm, cyclone, 1978	171	Cornell Medical Index Health Questionnaire (CMI)	1 month post	84 %
Cohort study						
Amstadter et al. 2009 [24]	Vietnam (medium)	Storm, Typhoon Xangsan, 2006	797	Pre: SRQ; Post: Structured Clinical Interview for DSM-IV (modified)	3 months post	2.2 %

Explanation: ^**a**^Human Development Index category

Additionally investigated risk factors were: poor health, high exposure, prior traumatic events [24], superior age, destruction of the home, seeing dead bodies and seeing dead family members [33].

**Fig. 7:**
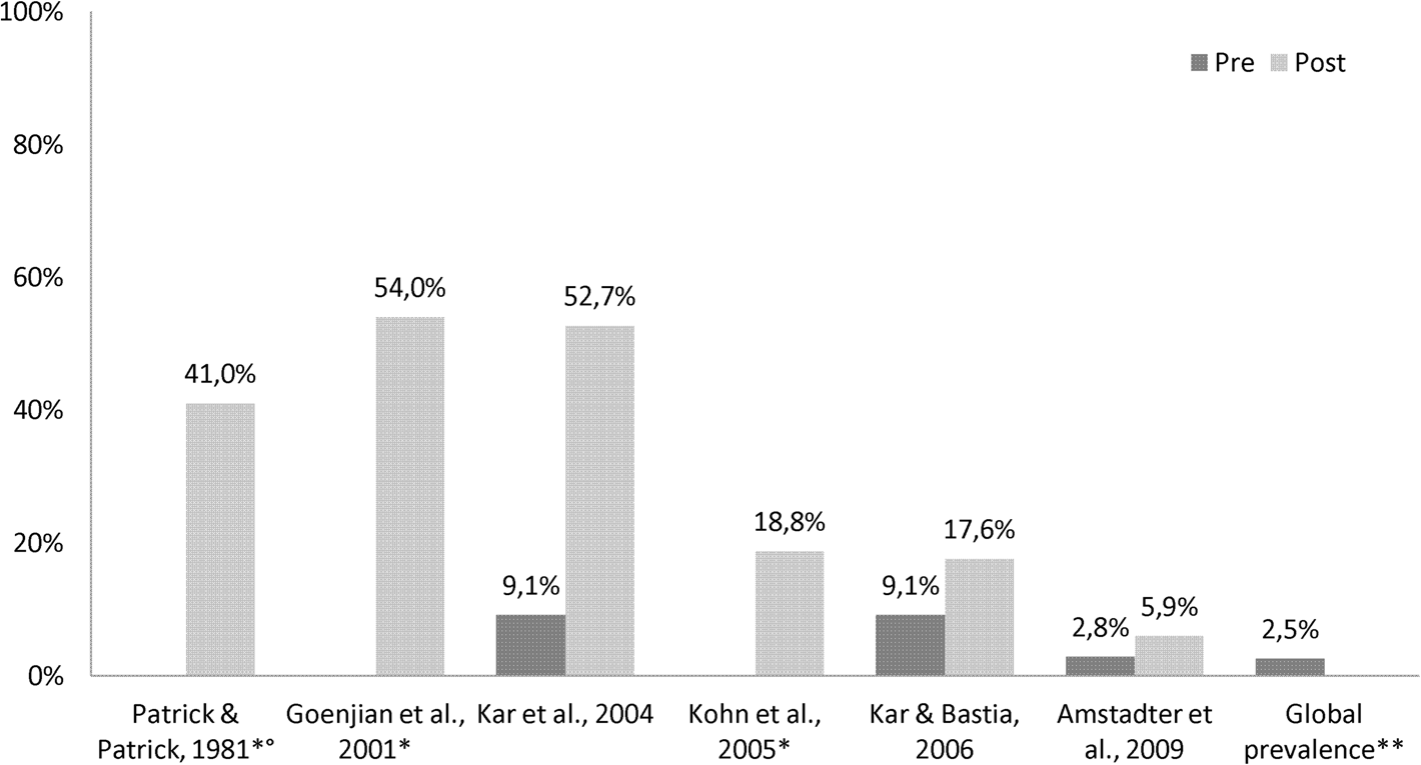
Pre/post analysis of depression prevalence in cross sectional and cohort° studies (*data not available; **according to GBD [19])

**Depressive disorder** prevalence rates (Table 5) differed throughout the studies and the global prevalence rate (2.6 % [19]) is additionally reported. All identified post-disaster rates were higher compared to the one in the non-exposed (Fig. 7).

**Table 5:** Results of depression prevalence rates in individual studies (total *n* = 2574)

Study (author/year)	Country (HDI ^a^)	Event/year	N	Assessment tool	Time point measured	Depression prevalence
Cross sectional studies						
Goenjian et al. 2001 [25]	Nicaragua (medium)	Storm, Hurricane Mitch, 1998	158 students	Depression Self-Rating Scale (DSRS)	6 months post	81 %, 51 %, 29 %^b^
Kar et al. 2004 [33]	India (medium)	Storm, super-cyclone, 1999	540	HADS & SRQ	5 months post	52.7 %
Kar & Bastia 2006 [34]	India (medium)	Storm, super-cyclone, 1999	108 students	Clinical examination & MINI-KID	14 months post	17.6 %
Kohn et al. 2005 [44]	Honduras (medium)	Storm, Hurricane Mitch, 1998	800	DSM-IV/ICD-10 Symptom checklist	2 months post	19.7 %, 17.7 %, 18.8 %^c^
Patrick & Patrick 1981 [38]	Sri Lanka (high)	Storm, cyclone, 1978	171	CMI	1 month post	41 %
Cohort study						
Amstadter et al. 2009 [24]	Vietnam (medium)	Storm, Typhoon Xangsan, 2006	797	Pre: SRQ; Post: Structured Clinical Interview for DSM-IV	3 months post	5.9 %

Explanation: ^a^Human Development Index category;^b^ 3 differently affected cities; ^c^3 age groups

Four studies additionally investigated factors that contributed to the risk of suffering from depressive disorder. They identified a poor health status, prior traumatic events, high exposure [24], female sex [24, 34], death of family member [25], damage to the house or valuables, starving, seeing dead bodies and unemployment [33].

## Discussion

### PTSD

Disaster experiences are stressors, e.g. loss of a child, seeing a person getting injured or die. Most of the included studies describe those losses. Caldera et al. reported the death of 2000 Nicaraguans during a hurricane and the homelessness of more than 10,000 [26]; Huang et al. reported the death of 4150 and the displacement of more than 18 million people during a flood in China [27]. Rubonis & Bickmann, in reviewing 39 disaster studies, found that the global rate of psychopathology increased by approximately 17 % [35]. They found that psychological morbidity tends to affect 30–40 % of the disaster population within the first year. Two years after the event this level decreases but a persistent burden of disease was expected to remain chronicized.

The identified PTSD prevalence rates ranged from 2.6 % [24] after a typhoon in Vietnam up to 90 % [25] in students of the most severely affected Nicaraguan city. Possible reasons for this span are the variety in instruments, time points of measuring, included populations, disaster type, and study characteristics. The unidirectional elevation indicates that there is a true effect (PTSD increase). Especially for PTSD very few reference data were available. This deficiency was met by introducing the global prevalence rate in order to interpret the findings. This rate must be interpreted carefully, since it is a weighed and global measure.

Furthermore, the assessment tools must be compared cautiously. A post-traumatic reaction (like PTSD) is evidently accessed via instruments that explicitly refer to the disaster as stressor (e.g. IES). Several studies applied other instruments (e.g. SRQ) where the stressor is not assessed. Additionally, most assessment tools have been developed in a Western context. Also, the cultural fit of those more Western diagnoses might not necessarily apply for cultures of low income countries [24, 36].

### Injury

The highest injury rates were reported after storms in Grenada and China (35.7 and 37.9 %). The Grenadian report is based on the analysis of hospital records of less than 200 patients which raises the possibility of a sample selection bias. The report from China comprised more than 3000 individuals; the study was conducted a few days after the disaster had occurred. The study reporting the lowest injury rate (1.7 %) was conducted in infants (9–24 months) in Jamaica, 1988 [37].

### Anxiety disorder

The highest post-disaster rate identified in this review (84 %) (after a cyclone in Sri Lanka in 1978 [38]) might be explained by several aspects: early elevation of data (one month post-disaster), and the old age of the study - the assessment tools might be more accurate nowadays. The lowest reported post-disaster rate indicated a slight decline (pre/post difference: −0.4 %) (after a typhoon in Vietnam in 2006 [24]). There are several explanations: the study scored the lowest rates in each investigated outcome (PTSD, Major Depressive Disorder (MDD), General Anxiety Disorder (GAD)) compared to the other studies, and the described storm was not as devastating as the other disasters. An indicator for this is the low number of deaths (72), compared to 10,000 deaths during cyclone in India and 4150 during a flood in China. Additionally, the local infrastructures might differ, here it was reported that a successful evacuation took place.

### Depressive disorder

Depression rates ranged from 5.9 % (after the typhoon in Vietnam in 2006 [24]) to 81 % (after a Hurricane 1998 in Nicaragua [25]). The latter high rate might be caused by the fact that this study exclusively investigated students (who are more vulnerable [34]); by the stratification of three unequally stricken regions (other studies might not have assessed the most severely affected regions); and by the severity of the disaster claiming 4000 deaths, 500,000 displacements, and generally affecting more than two million people.

### Research in developing countries

Compared to high income countries, there is only a small amount of studies conducted in the global South. Very little of this research is on extreme weather events and particularly little on psychopathology [39]. The methodological insufficiencies of the current disaster literature from these countries include that sample selection is often not conducted in a representative manner and that there are no comparison groups [40].

There is a general lack of data in these countries so that in many cases health and mortality is described to be accessed via self-reporting [33]. Relying on self-reported health measures and merely pre-disaster information is a major limitation. Conservatively seen, several included measures do describe the subjective health rather than verified morbidity. Disaster studies are mostly conducted under extremely difficult conditions; there is no ideal setting for undertaking such study. The affected area is usually wide-ranging, the exposure is distributed unevenly, and some parts are most likely not accessible. The target population is disaster-stricken and might not be willing or able to answer comprehensive questionnaires.

Additionally, several assessment and measuring tools have been developed and validated in a Western context and might not ideally reflect the burden of disease in the less developed parts of the world [24, 36]. Thus the cultural fit of those more Western diagnoses might not necessarily apply for cultures of low income countries and the comparison to global PTSD rates should be interpreted with caution. There is a need to identify individual predictors that are culture specific as e.g. a PTSD diagnosis is criticized for not having cross–cultural validity [30]. No disaster on the African continent was identified, although, numerous disasters did occur there (Table 6) [41]. One reason for this under-reporting might be poverty. Most African countries are found in the lowest HDI category. Poverty is connected to weak local infrastructure (e.g. education, health services) and therefore very little data are accumulated [3]. This is supported by the fact that only two included studies are from countries of low human development.

**Table 6:** Effects of reported natural disasters (1900–2013) according to continent

Continent	Occurrence	Persons dead	Persons injured	Persons homeless	Total damage (in 1,000 USD)
Africa	1,422	879,837	42,786	7,694,237	14,338,143
Americas	2,591	237,128	1,935,341	7,331,807	837,539,590
Asia	3,925	17,784,181	2,590,169	128,323,963	643,657,316
Europe	1,307	1,373,994	53,501	1,967,437	270,889,910
Oceania	463	4,175	6,562	374,990	45,846,105

Explanation: category of natural disasters comprising subgroups of climatological, hydrological, and meteorological disasters (including drought, extreme temperature, flood, mass movement (wet), storm)

Neither studies on droughts nor heatwaves were identified. Most of the heat-related disasters occur in Africa [4]. Additionally, heatwaves and droughts are *creeping processes* - it is hard to identify the beginning, end and thus to collect data.

The growing number of extreme weather events leads to an increase in displacement, as reported in most included studies. Reasons for climate related migration have increased in the past decade [5]. The escape of individuals from their home country due to environmental disturbances is not yet embraced by the leading definition of a refugee provided by the UN High Commissioner for Refugees.

### Strengths and limitations of this review

The non-feasibility of conducting a meta-analysis results from the strong study heterogeneity. The search was limited to published articles and a number of selected sources. Prospective investigations might also search leading documents of e.g. UNFCCC or IPCC and grey literature in order to address the under-reporting from African countries and that of droughts and heatwaves.

The main strengths are the broad and effective search strategy as well as the work of two independent reviewers and their excellent level of agreement. Another strong point is the total of 70,842 included individuals and the overall moderate study quality. Even though there is much variation within the prevalence rates, a consistent increase in outcomes is found.

## Conclusion

Further gain in knowledge is: the confirmation of an under-reporting of certain disaster types and from certain regions, and a strong heterogeneity in measuring mental health outcomes.

### Implications for practice

Public Health decision makers are encouraged to both act now and address adaptation strategies in the long run. These should encompass: the establishment of strong health infrastructures, empowering communities to achieve effective disease surveillance, acquisitions and training of extra personnel, and implementation of disaster communication infrastructure. Guidelines of global health organizations (e.g. of WHO’s Inter-Agency Standing Committee) should be emphasized. In order to sustainably meet the needs of disaster affected populations, the detection of regions with increased risk, outlining of roles that actors will play in case of an emergency, training of responders, and the identification of vulnerabilities should be enhanced. Preparedness also includes the solid (re-)construction of health care facilities, infrastructure, as well as water and sanitation systems in which cultural and gender aspects should be carefully considered.

### Implications for decision makers

Previously, legal acts have been developed after disasters occurred in order to prevent repetitive harm. Several codes of conduct have been established which are, however, non-binding and therefore weak. The international community should aim at developing and adhering to measures that are preventive, fair, and future-oriented. Decision makers at country and regional level should encourage the improvement of mental health care infrastructure. Most countries are dependent on external assistance to meet post-disaster health needs. Due to national or ethical strife towards neighboring countries, conflicting political interests or poor coordination, many disasters have not been successfully dealt with. External support often does not match the local need which is especially true for the existing national mental health care systems, which do not meet the demand of post-disaster mental health problems. Thus, recommendations for required international assistance include, that assistance should involve partners who work in ways that are complementary to each other, engage the affected community, plus be evidence-based and transparent.

This review shows that the growing number of extreme weather events also leads to an increase in displacement of thousands of individuals. A range of additional health issues are associated with dislocation. Currently, about 51.2 million people worldwide are displaced, approximately 86 % find refuge in developing countries [10]. Many of them have not been able to return to their home countries for decades. The number of involuntary migrants is expected to increase and hence, requires a human rights-based response. This should include the development of adjustment instruments which should also incorporate financing plans both at national level as well as on behalf of the international community, plus the provision of a refugee status for those who fled their country due to environmental damage.

### Implications for research

There is the difficulty of establishing causation in a non-experimental design. Better health measures, stronger epidemiological designs, dose of exposure investigations, and follow-up assessments, providing long-term data are needed. Few databases on hazards and climate conditions have successfully been established (e.g. *UNFCCC Local coping strategies database*) and should expand. Overall, the collection of data (as the basis for scientific output) and the establishment of disease monitoring and early warning systems is encouraged, with the latter two being also of great importance for decision makers.

## Additional files

Additional file 1: PRISMA checklist. (DOC 63 kb) Additional file 2: Electronic search strategy for Medline database. (DOCX 13 kb)
