# Supplementary material for: Extreme weather events in developing countries and related injuries and mental health disorders - a systematic review
Source: BMC Public Health. 2016 Sep 29;16:1020. doi: 10.1186/s12889-016-3692-7 (PMC5041325; doi:10.1186/s12889-016-3692-7)
Supplement: Additional file 2: — Electronic search strategy for Medline database. (DOCX 13 kb) [file 12889_2016_3692_MOESM2_ESM.docx]

Additional file 2. Electronic search strategy for Medline database

((developing countries.mp. OR Developing Countries/ OR Poverty/ OR Rural Population/) AND (weather.mp. OR weather/ OR exp Climate/ OR climate change/ OR global warming/ OR Disasters/ OR humidity/ OR rain/ OR floods/ OR temperature/ OR hot temperature/ OR droughts/ OR extreme heat/ OR tornadoes/ OR wind/ OR air movements/ OR cyclonic storms/) AND (Stress Disorders, PostTraumatic/ OR ptsd.mp. OR posttraumatic stress disorder.mp.OR "wounds and injuries"/ OR amputation, traumatic/ OR drowning/ OR heat exhaustion/ OR heat stroke/ OR multiple trauma/ OR "sprains and strains"/ OR vascular system injuries/ OR mortality/ OR "cause of death"/ OR fatal outcome/ OR survival rate/ OR exp morbidity/ OR injury.mp. OR wound.mp. OR trauma.mp. OR damage.mp. OR Accidents/ OR Fractures, Bone/ OR anxiety/ OR exp anxiety OR disorders/ OR anxiety disorder.mp. OR panic disorder/ OR phobic disorders/ OR Obsessive-Compulsive Disorder/ OR behavioral symptoms/ OR depression/ OR depressive disorder/ OR depressive disorder, major/ OR Cardiovascular Diseases/ OR Myocardial Infarction/ OR stroke/ OR Brain infarction/ OR heart attack.mp. OR Coronary Disease/ OR Atrial Fibrillation/ OR Heart Failure/ OR hypertension/ OR myocardial ischemia/ OR peripheral vascular diseases/) AND (Epidemiologic studies/ OR Exp case control studies/ OR Exp cohort studies/ OR Case control.tw. OR (cohort adj (study or studies)).tw. OR Cohort analy$.tw. / OR (Follow up adj (study or studies)).tw. OR (observational adj (study or studies)).tw. OR Longitudinal.tw. OR Retrospective.tw. OR Cross sectional.tw. OR Cross-sectional studies/))
